# Dual inhibition of ATR and ATM potentiates the activity of trabectedin and lurbinectedin by perturbing the DNA damage response and homologous recombination repair

**DOI:** 10.18632/oncotarget.8292

**Published:** 2016-03-23

**Authors:** Michelle Lima, Hana Bouzid, Daniele G. Soares, Frédéric Selle, Claire Morel, Carlos M. Galmarini, João A. P. Henriques, Annette K. Larsen, Alexandre E. Escargueil

**Affiliations:** ^1^ Cancer Biology and Therapeutics, Centre de Recherche Saint-Antoine, Institut National de la Santé et de la Recherche Médicale (INSERM) UMR 938, Institut Universitaire de Cancérologie (IUC), Université Pierre et Marie Curie (UPMC), Sorbonne Universités, Paris, France; ^2^ Departamento de Biofísica, Centro de Biotecnologia, Universidade Federal do Rio Grande do Sul, Porto Alegre, Brazil; ^3^ Medical Oncology Department, Hospital Tenon, Public Assistance Hospitals of Paris (AP-HP), Alliance Pour la Recherche en Cancérologie (APREC), Paris, France; ^4^ Cell Biology Department, PharmaMar, Poligono Industrial La Mina, Colmenar Viejo, Madrid, Spain; ^5^ Instituto de Biotecnologia, Universidade de Caxias do Sul, Caxias do Sul, RS, Brazil

**Keywords:** DNA double strand breaks, DNA alkylators, DNA replication, homologous recombination, checkpoint abrogators

## Abstract

Trabectedin (Yondelis^®^, ecteinascidin-743, ET-743) is a marine-derived natural product approved for treatment of advanced soft tissue sarcoma and relapsed platinum-sensitive ovarian cancer. Lurbinectedin is a novel anticancer agent structurally related to trabectedin. Both ecteinascidins generate DNA double-strand breaks that are processed through homologous recombination repair (HRR), thereby rendering HRR-deficient cells particularly sensitive. We here characterize the DNA damage response (DDR) to trabectedin and lurbinectedin in HeLa cells. Our results show that both compounds activate the ATM/Chk2 (ataxia-telangiectasia mutated/checkpoint kinase 2) and ATR/Chk1 (ATM and RAD3-related/checkpoint kinase 1) pathways. Interestingly, pharmacological inhibition of Chk1/2, ATR or ATM is not accompanied by any significant improvement of the cytotoxic activity of the ecteinascidins while dual inhibition of ATM and ATR strongly potentiates it. Accordingly, concomitant inhibition of both ATR and ATM is an absolute requirement to efficiently block the formation of γ-H2AX, MDC1, BRCA1 and Rad51 foci following exposure to the ecteinascidins. These results are not restricted to HeLa cells, but are shared by cisplatin-sensitive and -resistant ovarian carcinoma cells. Together, our data identify ATR and ATM as central coordinators of the DDR to ecteinascidins and provide a mechanistic rationale for combining these compounds with ATR and ATM inhibitors.

## INTRODUCTION

Trabectedin (Yondelis®, ecteinascidin-743, ET-743) is a marine-derived natural product that is approved for treatment of patients with advanced soft tissue sarcoma and relapsed platinum-sensitive ovarian cancer [1]. Lurbinectedin (PM01183) is a novel ecteinascidin (ET) derivative in clinical development [2]. Lurbinectedin is structurally similar to trabectedin except for a tetrahydroisoquinoline present in trabectedin that is replaced by a tetrahydro β-carboline in lurbinectedin [3]. This structural variation is accompanied by important modifications of the pharmacokinetic and pharmacodynamic properties in cancer patients although the preclinical activities of lurbinectedin remain close to those observed for trabectedin [4,5].

Due to their original mechanism of action, trabectedin and lurbinectedin are associated with an unusual pattern of sensitivity in DNA repair-deficient cells [1]. Several studies have shown that in contrast to other DNA-targeted anticancer agents, TC-NER-deficient cells are 2 to 10 times more resistant to trabectedin and lurbinectedin [5–10]. It was also shown that homologous recombination repair (HRR), but not Non-Homologous End Joining (NHEJ), is important for trabectedin and lurbinectedin, since HRR-deficient cells were 50 to 100 times more sensitive to these drugs. The lack of HRR was associated with the persistence of unrepaired DSBs during the S phase of the cell cycle and apoptosis [5,11,12]. Importantly, the unique sensitivity of cells deficient in HRR has been confirmed in the clinic [13–15]. Interestingly, although HRR deficiency has proven relevant for both trabectedin and lurbinectedin [5], no strategy has been evaluated to inhibit this repair pathway although it would likely improve the activity of the ecteinascidins (ETs) by mimicking HRR deficiency. Moreover, inhibition of the cell cycle checkpoints that are activated in response to trabectedin might also prove useful in order to increase drug efficacy [16,17].

The major regulators of the DNA damage response (DDR) are two phosphatidyl inositol 3-kinase-like kinases (PIKKs), ataxia-telangiectasia mutated (ATM) and ATM and RAD3-related (ATR) [18]. ATM initiates the cellular response to DSBs. ATM is activated through autophosphorylation of the Ser1981 residue and activates the distal transducer kinase, Chk2 [18–20]. The primary function of ATR is to monitor DNA replication and to regulate the repair of damaged replication forks [18,21]. ATR is recruited by the ATR-interacting protein (ATRIP) to regions of replication protein A (RPA)-coated stretches of single-stranded DNA (ssDNA) that are generated by decoupling of helicase and polymerase activities at stalled replication forks [22–24]. Once activated, ATR preferentially phosphorylates the distal kinase, Chk1 [18, 21]. Both ATM/Chk2 and ATR/Chk1 pathways converge to inactivate members of the Cdc25 phosphatase family, which drives dividing cells through the cell cycle [25]. In addition to their specific substrates, ATM and ATR also share common ones, like the histone variant H2AX and the 32-kDa subunit of human RPA (RPA32). RPA32 phosphorylation, catalyzed by the PIKKs family as well as CDKs, plays an important role in stabilizing DNA replication forks and in promoting HRR in response to replication arrest [26,27]. RPA32 phosphorylation occurs at the site of damage where it marks the sites of DNA damage or DNA stress [28]. The phosphorylation of the histone variant H2AX leading to the formation of the so-called γ-H2AX might serve as docking sites for DNA damage/repair proteins, including MDC1, 53BP1 and BRCA1, and functions to promote DSB repair and genome stability [25,29,30]. In this process, the binding of MDC1 to H2AX acts as the first step where γ-H2AX-associated MDC1 recruits additional activated ATM, thereby establishing a positive feedback loop leading to γ-H2AX expansion along the DNA [31–33]. Importantly, MDC1 is also involved in ATR-dependent Chk1 activation by promoting accumulation of TopBP1 at stalled replication forks thus facilitating the efficient activation of ATR kinase activity [34]. In addition to recruiting MDC1, γ-H2AX helps recruiting BRCA1, a central constituent of HRR [30,35]. BRCA1 then promotes the recruitment of BRCA2 which in turn favors the recruitment of RAD51 for homologous recombination [35].

In this study, we characterize the DNA damage response to trabectedin and lurbinectedin in HeLa cells. Our results show that both compounds activate the ATM/Chk2 and ATR/Chk1 pathways simultaneously which is accompanied by the formation of BRCA1 and Rad51 foci. Interestingly, the pharmacological inhibition of either Chk1/2 (AZD7762), ATR (VE-821, AZ20) or ATM (KU-60019) kinase is not accompanied by any significant increase in the cytotoxicity of trabectedin or lurbinectedin. In contrast, simultaneous inhibition of both ATM and ATR strongly potentiates the activity of the ETs. To explain this phenomenon, we show that concomitant inhibition of both ATR and ATM is an absolute requirement to efficiently block the formation of γ-H2AX, MDC1, BRCA1 and Rad51 foci suggesting a redundant or complementary function of the ATM and ATR pathways in the processing of ET-induced DSBs. Importantly, these results are not restricted to HeLa cells, but can also be extended to cisplatin-sensitive and -resistant ovarian cancer cell lines. Together, our data identify ATR and ATM as central coordinators of the DDR to trabectedin and lurbinectedin and provide a mechanistic rationale for combinations of these compounds with dual ATR and ATM inhibitors.

## RESULTS

### Trabectedin and lurbinectedin induce both ATM- and ATR-dependent DNA damage response pathways

**Figure 1:**
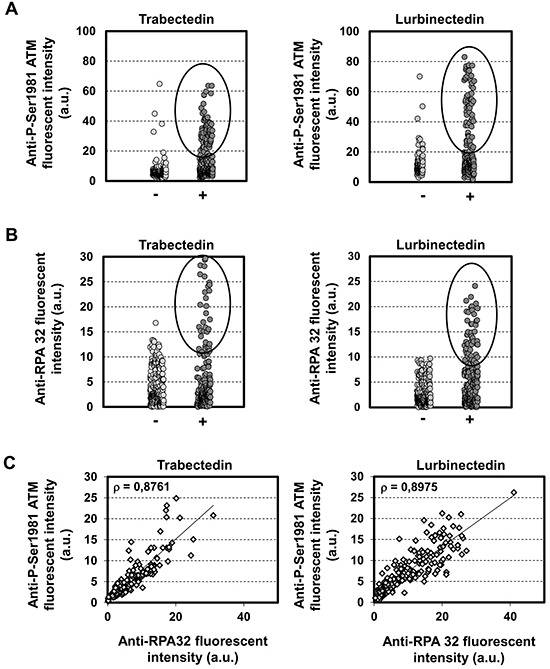
Trabectedin and lurbinectedin activate both ATM and ATR pathways **A.** HeLa cells were mock-treated or exposed to 20 nM trabectedin (left panel) or lurbinectedin (right panel) for 1 hour followed by 6 hours post-incubation in drug-free media. Cells were then processed for immunolabeling with an antibody directed against Ser1981-phosphorylated ATM. Fluorescence intensities in individual cells were quantified by Metamorph analysis and are indicated in arbitrary units (a.u.). **B.** Same as above except than the cells were pre-permeabilized with ice-cold CSK-lysis buffer to remove the soluble fraction of RPA32 before processing for immunolabeling with an antibody directed against RPA32. **C.** Same as above except than cells were processed for simultaneous staining of Ser1981-phosphorylated ATM and RPA32. ρ denotes the correlation coefficient between the intensities of Ser1981-phosphorylated ATM and RPA32.

**Figure 2:**
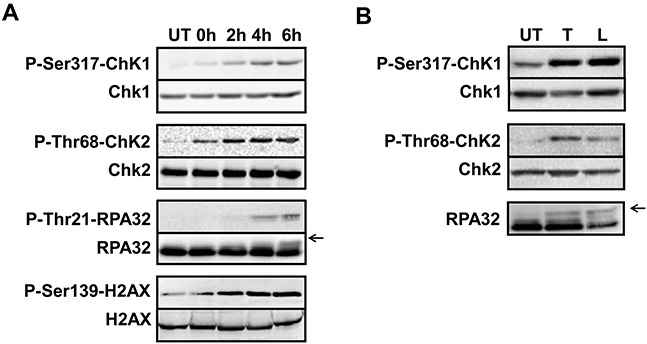
Both trabectedin and lurbinectedin induce the DNA damage response **A.** HeLa cells were mock-treated (UT) or incubated for 1 hour with trabectedin (20 nM) followed by 0, 2, 4, 6 hours post-incubation in drug-free media as indicated. Total protein extracts were prepared and analyzed by immunolabeling with antibodies directed against Ser317-phosphorylated Chk1, Thr68-phosphorylated Chk2, Thr21-phosphorylated RPA32 and Ser139-phosphorylated H2AX. Total Chk1, Chk2, RPA32 and H2AX were used as loading controls. **B.** HeLa cells were either untreated (UT) or incubated for 1 hour with 20 nM trabectedin (T) or lurbinectedin (L) followed by 6 hours post-incubation in drug-free media. Total protein extracts were prepared and analyzed by immunolabeling with antibodies directed against Ser317-phosphorylated Chk1, Thr68-phosphorylated Chk2 and RPA32. Total Chk1 and Chk2 were used as loading controls. On each panel, arrows indicate the main phosphorylated forms of RPA32.

Previous studies indicate that trabectedin induces replication-dependent DSBs [11]. To identify the key factors needed for the DDR to trabectedin and lurbinectedin, we first determined the activity of ATM. Immunofluorescence microscopy was used to determine the activation of ATM, as measured by ATM autophosphorylation of Ser1981 after 1 hour exposure to 20 nM trabectedin (Figure 1A, left panel) or lurbinectedin (Figure 1A, right panel) followed by 6 hours post-incubation in drug-free media. The results show that both compounds induce the autophosphorylation of ATM, compared to untreated control cells (Figure 1A). Interestingly, only a subset of cells shows autophosphorylation of ATM (Figure 1A). These data are in agreement with previous reports demonstrating that ATM plays a role in the processing of replication-dependent DSBs induced by trabectedin [36]. Coherent with the results for ATM, both trabectedin and lurbinectedin treatments lead to the activation of Chk2 through phosphorylation on Thr68 (Figure 2). Together, our results indicate that the ATM/Chk2 pathway is activated in response to trabectedin and lurbinectedin.

To determine whether the ATR/Chk1 pathway also plays a role in the processing of trabectedin- or lurbinectedin-induced DNA lesions, we performed immunofluorescence microscopy to visualize chromatin recruitment of RPA32, one of the three RPA subunits (Figure 1B). RPA-coated single-stranded DNA (ssDNA) regions are required for recruitment of the ATR-ATRIP complex to damaged sites [22–24]. Our results show that 1 hour exposure to 20 nM trabectedin (Figure 1B, left panel) or lurbinectedin (Figure 1B, right panel) followed by 6 hours post-incubation in drug-free media is accompanied by strong chromatin recruitment of RPA32. Remarkably, the trabectedin- and lurbinectedin-induced RPA foci were mostly detected in cells where ATM was autophosphorylated (Figure 1C) suggesting that both pathways are activated simultaneously following exposure to the ETs. Chromatin recruitment of RPA32 was accompanied by rapid activation of ATR as indicated by the formation of phosphorylated Chk1 on the Ser317 residue (Figure 2). Interestingly, RPA32 as well as the H2AX histone variant are phosphorylated in response to trabectedin (Figure 2A and Supplementary Figures S1A and S1B) or lurbinectedin (Figure 2B and Supplementary Figures S2A and S2B). This suggests that the damaged replicative sites are quickly recruiting proteins capable of stabilizing the replication fork and repairing the DSBs. In agreement, we show that BRCA1 is recruited to the chromatin following exposure to trabectedin (Supplementary Figure S1C) or lurbinectedin (Supplementary Figure S2C).

### Combination of trabectedin and lurbinectedin with checkpoint abrogators

**Figure 3:**
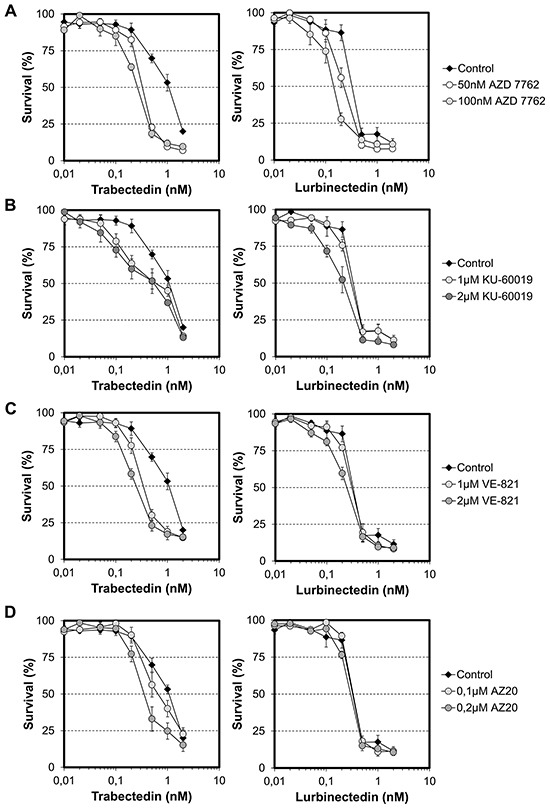
Effect of single checkpoint abrogators on the cytotoxic activities of trabectedin and lurbinectedin HeLa cells were exposed for 1 hour to the indicated concentrations of checkpoint abrogators (**A.** AZD7762; **B.** KU-60019; **C.** VE-821; **D.** AZ20) before addition of either trabectedin (left panels) or lurbinectedin (right panels) at the indicated concentrations. Cells continuously exposed to trabectedin or lurbinectedin alone were included as control. AZD7762, KU-60019, VE-821 and AZ20 have no effects on HeLa cells growth when used alone up to 100 nM, 2 μM, 2 μM and 0.2 μM, respectively. Standard deviations (SD) are indicated by error bars and are indicated when they exceed symbol size.

Previous reports have shown that individual checkpoint abrogators enhance the efficacy of DNA-targeting anticancer drugs as well as of radiotherapy [37–40]. To establish if this approach is also valid for the ETs, the influence of pharmacological concentrations of Chk1/Chk2 (AZD7762, 50 and 100 nM), ATM (KU60019, 1 and 2 μM) or ATR (VE-821, 1 and 2 μM; AZ20, 0.1 and 0.2 μM) inhibitors on the cytotoxicity of the ETs was determined. However, the presence of a single checkpoint abrogator had only modest influence on the cytotoxicity of the ETs. Indeed, the activity of trabectedin was increased 4-, 2-, 4- and 3-fold by AZD7762, KU60019, VE-821 or AZ20, respectively (Figure 3, left panels). Similarly, the activity of lurbinectedin was only marginally increased when cells were co-incubated with AZD7762 (Figure 3, right panels). Together, these results show that the classical strategies of using a single checkpoint abrogator as chemo-sensitizer do not apply to the ETs and suggest either no role or, alternatively, functional overlap of the ATM and ATR pathways in the processing of ETs-induced DNA lesions.

### Influence of dual ATM and ATR inhibition on the cytotoxicity of trabectedin and lurbinectedin

**Figure 4:**
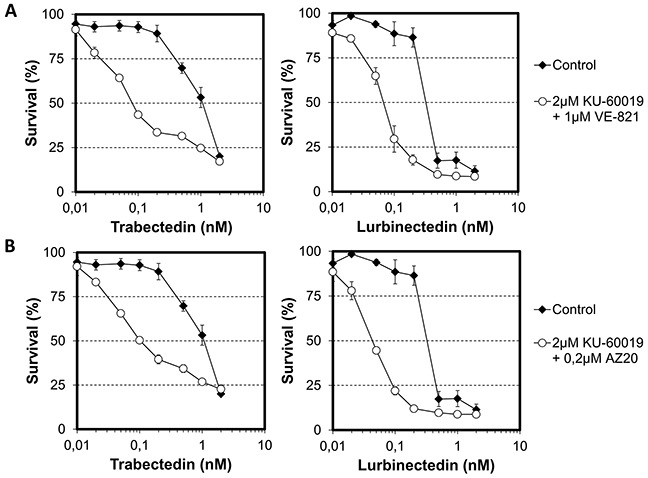
Influence of combinations of checkpoint abrogators on the cytotoxic activities of trabectedin and lurbinectedin **A.** HeLa cells were first exposed for 1 hour to either no drug (black diamond) or a combination of 2 μM KU-60019 and 1 μM VE-821 (white circle) before addition of either trabectedin (left panel) or lurbinectedin (right panel) at the indicated concentrations. **B.** HeLa cells were first exposed for 1 hour to either no drug (black diamond) or a combination of 2 μM KU-60019 and 0.2 μM AZ20 (white circle) before addition of either trabectedin (left panel) or lurbinectedin (right panel) at the indicated concentrations. Both combinations of checkpoint abrogators, that is 2 μM KU-600019 with 1 μM VE-821 and 2 μM KU-600019 with 0.2 μM AZ20 have minor cytotoxic activity (<IC_20_) toward HeLa cells by themselves. SDs are indicated by error bars and are indicated when they exceed symbol size.

To distinguish between these two possibilities, we performed viability assays on HeLa cells that were treated with ETs in the absence or presence of dual ATM and ATR inhibition (with KU60019 and VE-821, respectively). We chose concentrations of checkpoint abrogators (2 μM KU60019 and 1 μM VE-821) with marginal toxicity toward HeLa cells (<IC_20_) when combined. Interestingly, although inhibition of ATM or ATR only moderately increased the cytotoxic activity of trabectedin (Figures 3B and 3C, left panels), dual inhibition of ATM and ATR potentiated the cytotoxicity of trabectedin 14-fold (Figure 4A, left panel). Similarly, although inhibition of ATM or ATR alone had no effect on the cytotoxicicity of lurbinectedin (Figures 3B and 3C, right panels), dual ATM and ATR inhibition markedly increased it (Figure 4A, right panel). Importantly, these observations were not limited to a specific type of cell cycle abrogator, since the combination of 2 μM KU60019 with 0.2 μM AZ20 also improved the cytotoxic activities of trabectedin and lurbinectedin by 11- and 8-fold, respectively (Figure 4B). These results strongly suggest that both ATM and ATR act in the signaling of ET-induced DNA damage and therefore, that both need to be inhibited in order to increase the cytotoxic activity of the ETs.

### Both ATM and ATR are involved in the initial steps of the DDR

**Figure 5:**
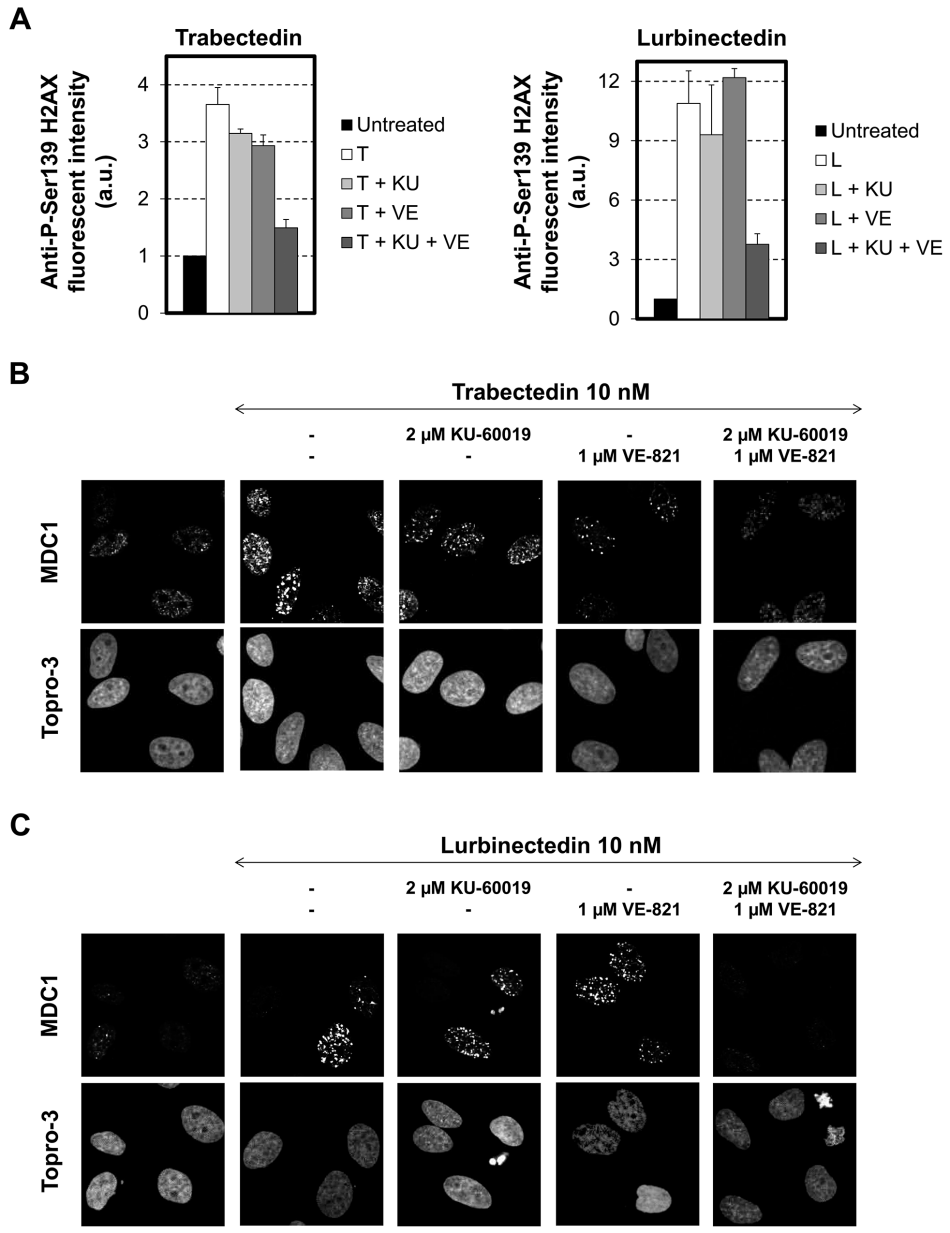
Influence of combinations of checkpoint abrogators on the phosphorylation of the histone variant H2AX and the focalization of MDC1 following exposure to trabectedin or lurbinectedin **A.** HeLa cells were exposed to 10 nM trabectedin (left panel, T) or lurbinectedin (right panel, L) for 1 hour in the absence (white columns) or presence of 2 μM KU-60019 (+ KU, light grey columns), 1 μM VE-821 (+ VE, medium grey columns) or a combination of 2 μM KU-600019 and 1 μM VE-821 (+ KU + VE, dark grey columns). This was followed by 24 hours post-incubation in the absence (white columns) or presence of 2 μM KU-60019 (+ KU, light grey columns), 1 μM VE-821 (+ VE, medium grey columns) or a combination of 2 μM KU-600019 and 1 μM VE-821 (+ KU + VE, dark grey columns). Cells were then processed for immunolabeling with an antibody directed against Ser139-phosphorylated H2AX. Untreated cells were used as a negative control (black columns). The fluorescence intensities in single cells were quantified by Metamorph analysis and are expressed in arbitrary units (a.u.). Data are represented as means +/− SD. **B.** (trabectedin) and **C.** (lurbinectedin), Same as above, except that cells were pre-permeabilized with ice-cold CSK-lysis buffer before fixation and immunolabeling with a MDC1-directed antibody. DNA was counterstained with Topro-3 fluorescent dye. MDC1 focalization was visualized by confocal microscopy.

To better characterize the molecular processes underlying the need for dual ATM/ATR inhibition to improve the activity of the ETs, we first determined the influence of 2 μM KU60019, 1 μM VE-821 or 2 μM KU60019 in combination with 1 μM VE-821 on the phosphorylation of histone H2AX following exposure to trabectedin or lurbinectedin (Figure 5A). Interestingly, our results show that the formation of γ-H2AX foci is, at the best, only moderately diminished in the presence of a single kinase inhibitor in response to the ETs. In clear contrast, dual inhibition of ATM and ATR was accompanied by a drastic reduction of γ-H2AX foci formation induced by trabectedin (Figure 5A, left panel) or lurbinectedin (Figure 5A, right panel). Accordingly, MDC1 chromatin recruitment and focalization was detectable when trabectedin- or lurbinectedin-treated cells were co-incubated in the presence of either KU60019 or VE-821 (Figure 5B and 5C) whereas the combination of both KU60019 and VE-821 completely inhibited the formation of MDC1 foci (Figure 5B and 5C). This observation was not limited to H2AX and MDC1, since RPA32 phosphorylation was also attenuated by dual, but not by single, inhibition of ATM or ATR (Supplementary Figure S3). It is interesting to note that single inhibition of either ATM or ATR generally has a more pronounced effect on trabectedin, compared to lurbinectedin, suggesting that the two compounds induce a similar, but not identical response. Together, these data suggest that both the ATM and the ATR kinase play a role in the initial DNA damage response to the ETs.

### Both ATM and ATR are required for the recruitment of HRR proteins

**Figure 6:**
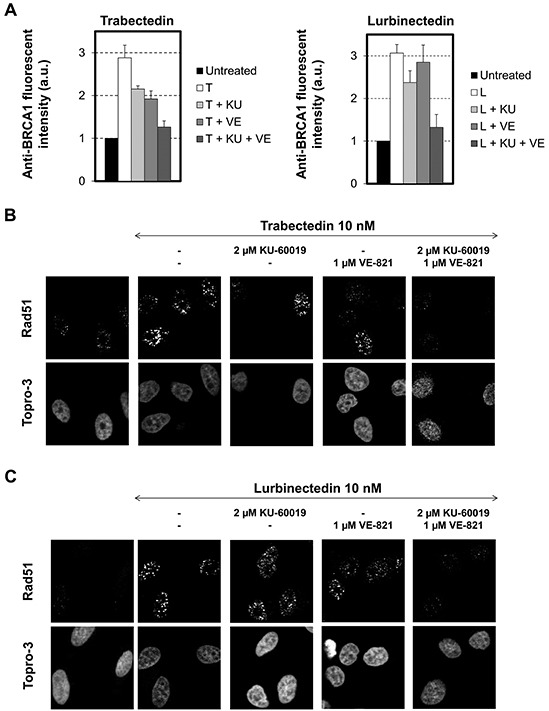
Influence of combinations of checkpoint abrogators on the focalization of BRCA1 and Rad51 induced by trabectedin or lurbinectedin **A.** HeLa cells were exposed to 10 nM trabectedin (left panel, T) or lurbinectedin (right panel, L) for 1 hour in the absence (white columns) or presence of 2 μM KU-60019 (+ KU, light grey columns), 1 μM VE-821 (+ VE, medium grey columns) or a combination of 2 μM KU-600019 and 1 μM VE-821 (+ KU + VE, dark grey columns). This was followed by 24 hours post-incubation in the absence (white columns) or presence of 2 μM KU-60019 (+ KU, light grey columns), 1 μM VE-821 (+ VE, medium grey columns) or a combination of 2 μM KU-600019 and 1 μM VE-821 (+ KU + VE, dark grey columns). Cells were then pre-permeabilized with ice-cold CSK-lysis buffer, fixed and immunolabeled with a BRCA1-directed antibody. Untreated cells were used as a negative control (black columns). The fluorescence intensities in single cells were quantified by Metamorph analysis and are expressed in arbitrary units (a.u.). Data are represented as mean +/− SD. **B.** (trabectedin) and **C.** (lurbinectedin), Same as above, except that cells were directly fixed and immunolabeled with a Rad51-directed antibody. DNA was counterstained with Topro-3 fluorescent dye. Rad51 focalization was visualized by confocal microscopy.

To determine if the inhibition of the early steps of the ETs-induced DNA-damage signaling is accompanied by a default in the recruitment of HRR proteins to the damaged DNA, we performed immunofluorescence microscopy to characterize the influence of ATM and ATR inhibition on the formation of BRCA1 and Rad51 foci (Figure 6). Again, we observed that the presence of a single kinase inhibitor only partly inhibited the formation of BRCA1 foci following trabectedin exposure (Figure 6A, left panel). In contrast, BRCA1 recruitment was not significantly influenced by ATM or ATR inhibition in response to lurbinectedin (Figure 6A, right panel) confirming the similar, but not fully identical, cellular response to the two ETs. In clear contrast, dual inhibition of both ATM and ATR almost completely inhibited the recruitment of BRCA1 to the chromatin following exposure to both trabectedin (Figure 6A, left panel) and lurbinectedin (Figure 6A, right panel). These results were not limited to BRCA1, since Rad51 focalization was also completely abrogated by dual, but not by single, inhibition of ATM and ATR (Figure 6B and 6C).

### Dual inhibition of ATM and ATR increases chromosome damage induced by trabectedin and lurbinectedin

**Figure 7:**
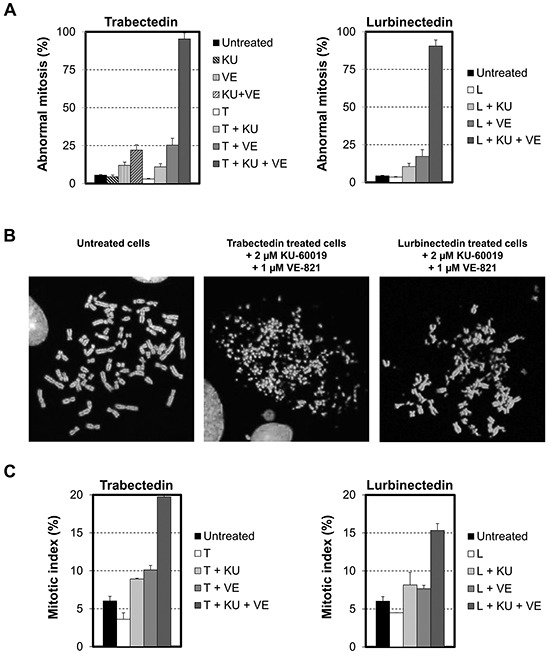
Influence of the combination of checkpoint abrogators on DSBs repair **A.** HeLa cells were exposed to 1 nM trabectedin (left panel, T) or lurbinectedin (right panel, L) for 1 hour in the absence (white columns) or presence of 2 μM KU-60019 (+ KU, light grey columns), 1 μM VE-821 (+ VE, medium grey columns) or a combination of 2 μM KU-600019 and 1 μM VE-821 (+ KU + VE, dark grey columns). This was followed by 24 hours post-incubation in the absence (white columns) or presence of 2 μM KU-60019 (+ KU, light grey columns), 1 μM VE-821 (+ VE, medium grey columns) or a combination of 2 μM KU-600019 and 1 μM VE-821 (KU + VE, dark grey columns). Cells were then processed for karyotype analysis. Untreated cells were used as a negative control (black columns). The left panel shows the influence on HeLa cells of 2 μM KU-60019 (KU, light grey dashed column), 1 μM VE-821 (VE, medium grey dashed column) or a combination of 2 μM KU-600019 and 1 μM VE-821 (KU + VE, dark grey dashed column) when they were given in the absence of ETs. Data are represented as mean +/− SD. **B.** Typical metaphase in untreated HeLa cells and cells treated for 1 hour with 1 nM of either trabectedin or lurbinectedin combined with a combination of 2 μM KU-600019 and 1 μM VE-821 and post-incubated for 24 hours in the presence of a combination of 2 μM KU-600019 and 1 μM VE-821. **C.** The mitotic index was determined on the microscopy slides used for karyotype analysis. Data are expressed as mean +/− SD.

Unrepaired DSBs may lead to chromosomal abnormalities. To determine the influence of checkpoint abrogators on the karyotype of ETs-treated cells, HeLa cells were exposed for 1 hour to a non-toxic concentration (1 nM) of either trabectedin or lurbinectedin in the presence or absence of 2 μM KU60019, 1 μM VE-821 or a combination of the two checkpoint abrogators. HeLa cells were then post-incubated in the presence or absence of checkpoint abrogators for 24 hours and their karyotype analyzed (Figure 7). In agreement with our previous findings, we show that single kinase inhibition slightly increased the chromosomal damage induced by trabectedin or lurbinectedin (Figure 7A). In clear contrast, dual inhibition of both ATM and ATR is accompanied by a striking increase in chromosome breakage induced by trabectedin (Figure 7A, left panel) as well as by lurbinectedin (Figure 7A, right panel). Importantly, this increase was well above the effects seen for the two checkpoint abrogators when they were given alone or in combination to cells in the absence of ETs (Figure 7A, left panel). Remarkably, all metaphases examined in cells treated with ETs in the presence of dual ATM and ATR inhibition showed extensive chromosome breakage (Figure 7B). Previous findings show that exposure to trabectedin or lurbinectedin induced cell cycle arrest in G2, most likely to allow time for DNA repair [5]. Accordingly, in our chromosome-spread experiments, we observed a slight decrease in the number of mitotic cells after treatment with the ETs (Figure 7C). In contrast, when cells were exposed to trabectedin or lurbinectedin in the presence of both ATM and ATR inhibitors, the fraction of mitotic cells increased from 3.5% to 20% and from 4% to 15%, respectively. In comparison, single kinase inhibition only partly replicated these results (Figure 7C). Importantly, VE-821 and KU60019 did not alter the fraction of mitotic cells by themselves (data not shown). Together, our findings show that the simultaneous inactivation of both ATM and ATR is necessary to increase the cytotoxic activities of the ETs acting through a potent and complete inhibition of the early DDR, on the recruitment of HRR proteins as well as on the subsequent G2/M checkpoint arrest resulting in the accumulation of deadly DSBs and mitotic catastrophe.

### Influence of dual ATM/ATR inhibition on the cytotoxic activities of trabectedin and lurbinectedin toward ovarian cancer cell lines

**Figure 8:**
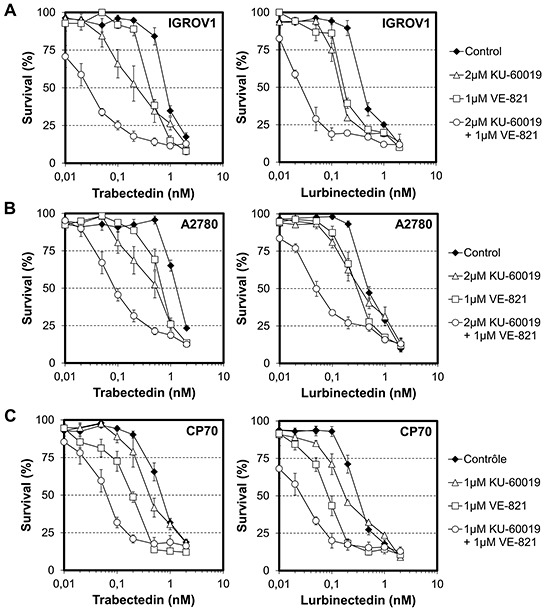
Influence of combinations of checkpoint abrogators on the cytotoxic activities of trabectedin and lurbinectedin toward ovarian cancer cell lines **A.** IGROV1 cells were first exposed for 1 hour to either no drug (black diamond), 2 μM KU-60019 (white triangle), 1 μM VE-821 (white square) or a combination of 2 μM KU-60019 and 1 μM VE-821 (white circle) before addition of either trabectedin (left panel) or lurbinectedin (right panel) at the indicated concentrations. The combination of 2 μM KU-600019 and 1 μM VE-821 had a minor effect (<IC_20_) on IGROV1 cells while 2 μM KU-600019 or 1 μM VE-821 alone had no toxicities. **B.** A2780 cells were first exposed for 1 hour to either no drug (black diamond), 2 μM KU-60019 (white triangle), 1 μM VE-821 (white square) or a combination of 2 μM KU-60019 and 1 μM VE-821 (white circle) before addition of either trabectedin (left panel) or lurbinectedin (right panel) at the indicated concentrations. The combination of 2 μM KU-600019 and 1 μM VE-821 as well as 2 μM KU-600019 or 1 μM VE-821 alone have no toxicity toward A2780 cells. **C.** A2780/CP70 cells were first exposed for 1 hour to either no drug (black diamond), 1 μM KU-60019 (white triangle), 1 μM VE-821 (white square) or 1 μM KU-60019 in combination with 1 μM VE-821 (white circle) before addition of either trabectedin (left panel) or lurbinectedin (right panel) at the indicated concentrations. The combination of 1 μM KU-600019 and 1 μM VE-821 has a minor effect (<IC_20_) on A2780/CP cells growth while either 1 μM KU-600019 or 1 μM VE-821 alone have no toxicities. SDs are indicated by error bars and are indicated when they exceed symbol size.

To confirm that our data might have some rapid clinical application, we applied our strategy to 3 different ovarian cancer cell lines. Remarkably, while ATM or ATR single inhibition increased the cytotoxic activity of trabectedin toward IGROV1 cells by 4- and 2-fold, respectively (Figure 8A, left panel, white triangle and white square), the combination of trabectedin with 2 μM KU60019 and 1 μM VE-821 strongly potentiated it 27-fold (Figure 8A, left panel, white circle). Similarly, while both ATM and ATR single inhibition improved the cytotoxic activity of lurbinectedin 3-fold each (Figure 8A, right panel, white triangle and white square), their combination markedly increases it 16-fold (Figure 8A, right panel, white circle). Importantly, these results were also found for A2780 cells as well as for their cisplatin-resistant A2780/CP70 counterparts (Figure 8B and 8C). Specifically, ATM and ATR dual inhibition increased the cytotoxic activities of trabectedin and lurbinectedin toward A2780 cells 13- and 10-fold, respectively, and 11-fold for both ETs toward A2780/CP70 cells. It is noteworthy that A2780/CP70 cells were more sensitive to dual kinase inhibition than the parental cells. Indeed, to reach a similarly low toxicity as for A2780 or IGROV1 cells (<IC_20_), A2780/CP70 cells had to be pre-incubated with 1 μM KU60019 and 1 μM VE-821 instead of 2 μM KU60019 and 1 μM VE-821. Taken together, our data demonstrate that combining ETs with dual ATM and ATR inhibition represent a promising approach to significantly improve the clinical efficacy of this unique class of DNA-targeting chemotherapeutics, in particular for patients with functional HRR.

## DISCUSSION

Although HRR deficiency has proven to be highly relevant for both trabectedin and lurbinectedin [5,11], no strategy has been evaluated so far to inhibit this repair pathway, although it would likely increase the antitumor activity of the ETs by mimicking HRR deficiency. This is likely because initial findings showed that ATM deficiency, which is believed to initiate the DDR following trabectedin exposure, only moderately increased the activity of both trabectedin and lurbinectedin in cellular models [5, 36]. In agreement, we here show that pharmacological inhibition of ATM by KU-60019, an ATP-competitive inhibitor, only marginally increased the cytotoxic activity of the ETs. The modest influence of ATM inhibition might be due to activation of alternative processes [41]. In agreement, we here report that ATM inhibition with KU-60019 only slightly inhibited γ-H2AX foci formation as well as chromatin recruitment of MDC1, BRCA1 and Rad51 following exposure to trabectedin or lurbinectedin. One might speculate that DNA-PK would be redundant with ATM as being the case for ionizing radiation [42]. However, in the case of trabectedin, DNA-PK is only involved in the processing of transcription-dependent, but not replication-dependent, DSBs. Furthermore, loss of DNA-PK has been associated with resistance, rather than increased sensitivity, to trabectedin thereby making DNA-PK a risky target [11,36].

Alternatively, one could imagine that ATR activation would be responsible for the modest influence of pharmacological inhibition or genetic loss of ATM. In agreement, our data show that the dual inhibition of both ATM and ATR is required to fully inhibit γ-H2AX foci formation and recruitment of HRR proteins 24 hours after exposure to trabectedin or lurbinectedin. Importantly, this is accompanied by a marked increase in the capacity of both ETs to induce chromosome damage and cell death. It is likely that ATR does not play an important role in the early phosphorylation of the histone variant H2AX since it has been reported that ATM inhibition leads to the almost complete loss of H2AX phosphorylation 6 hours after trabectedin exposure [36]. Preliminary data in our laboratory confirm that assumption (data not shown). This suggests that HRR starts at frank DSBs, leading to rapid ATM auto-phosphorylation and pathway activation. Accordingly, it has been suggested that by interfering specifically with the TC-NER process, trabectedin and lurbinectedin-induced DNA adducts are capable of forming ternary complexes that are not removed by the NER machinery, although the XPF/ERCC1 nuclease is able to cleave the strand opposite to the lesion thereby inducing SSBs [12,43]. Such SSBs could then be transformed into DSBs by the replication fork thus quickly activating the ATM pathway. Alternatively, the lack of early activation of the ATR pathway could lead to unstable replication forks leading to their collapse [36,44]. In agreement, both trabectedin and lurbinectedin form DNA adducts that stabilize double-stranded DNA (dsDNA) and functionally mimic covalent DNA cross-links thereby preventing the uncoupling of the helicase and polymerase activities needed for activation of ATR [3,43,45,46]. Interestingly, the role of ATM in dealing with replicative problems is not limited to ETs. In particular, it was shown that exposure to the hexavalent chromium [Cr(VI)] compounds results in generation of S phase-dependent DNA DSBs, which activate ATM independently of ATR [47]. Similarly, irofulven specifically induces the ATM/Chk2 signaling pathway in replicating cells [48,49]. More recently, it has been reported that low formaldehyde doses, by inducing chromatin perturbations, also causes a strong and rapid activation of ATM in human cells, which was ATR-independent and restricted to S-phase [50]. Together, these data show that ATM can deal with different types of replicative problems besides replicative stress. However, processing of stalled replication forks through either the FA pathway or replication fork regression might generate single-stranded DNA later capable of recruiting RPA thereby activating the ATR pathway [45,51,52]. The coexistence of different pathways to manage the stress induced by ETs is supported by our observation showing that both ATM and ATR pathways are activated within a single cell in response to ET-exposure. Interestingly, single inhibition of either ATM or ATR is likely to generate substrates capable of activating the remaining pathway. Indeed, the inhibition of ATM is likely to generate single-stranded DNA regions through the activation of endonucleases thereby activating the ATR pathway [53–55]. Conversely, the absence of ATR would promote replication fork collapse and activation of ATM [52]. Thus, the dual inhibition of both ATM and ATR is an absolute requirement to inhibit HRR and to potentiate ETs' activities.

Interestingly, a recent report has shown that the pharmacologic inhibition of ATR or ATM increased the response to ionizing radiation in human cervical, endometrial and ovarian carcinoma cell lines, with a further increase in ionizing radiation sensitization by coordinated inhibition of both kinases [56]. In contrast, selective inhibition of ATR, but not ATM, synergized with platinum in all three types of cellular models, while the combined inhibition of ATR and ATM does not enhance the response to platinum agents above that seen with the ATR inhibitor alone. These results, together with ours, demonstrate the need to precisely characterize the mechanism of action of each anticancer agent to establish new rationales and thereby to improve cancer patients' care. Until recently, clinical attempts to inhibit the DDR in order to ameliorate the activity of DNA-targeted agents were limited by the high general toxicity and lack of specificity of available compounds. However, new and selective inhibitors are currently under development and some have recently entered phase I clinical trials in combination with either radiotherapy or DNA-targeting agents [18,41]. Despite the fact that no ATM inhibitors are yet in clinical development and no ATR inhibitors have reached approval, the *in vitro* studies carried out to date clearly show that pharmacological inhibition of ATM and ATR has great potential in cancer therapy in combination with radiotherapy or certain chemotherapeutic drugs including trabectedin and lurbinectedin. One might speculate that inhibiting both ATM and ATR might have severe toxic effects on normal tissues when combined with DNA-targeting agents. Determining whether non-replicating cells are equally sensitized to ETs by dual inhibition of ATR and ATM than actively replicating tumor cells might be part of the answer. However, measuring the expression levels of ATR and ATM in tumors with functional HRR might also help to solve that issue. Tumors with low expression levels of ATR are indeed likely to respond to ETs when combined with ATM inhibitors while tumors with low expression levels of ATM are likely to respond to ETs when combined with ATR inhibitors. This approach might improve the therapeutic index of ETs on tumors with functional HRR by selectively targeting the tumor cells. Obviously, additional work on animal models is required to validate our combinations and to evaluate toxicities in a living context.

In summary, our findings demonstrate that pharmacological inhibition of either the Chk1/2, the ATR or the ATM kinase is not accompanied by any significant improvement of the cytotoxic activity of trabectedin or lurbinectedin. In clear contrast, dual ATM/ATR inhibition strongly potentiates the activity of both ETs against human cervical and ovarian carcinoma cells by efficiently blocking the formation of γ-H2AX, MDC1, BRCA1 and Rad51 foci following ET-exposure thereby resulting in extensive chromosome damage. Together, our data identify ATR and ATM as central coordinators of the DDR to trabectedin and lurbinectedin and provide a mechanistic rationale for combining these compounds with ATR and ATM inhibitors in future clinical trials.

## MATERIALS AND METHODS

### Chemicals

Trabectedin and lurbinectedin were provided by PharmaMar (Madrid, Spain). AZD7762 (http://www.selleckchem.com/products/AZD7762.html), AZ20 (http://www.selleckchem.com/products/az20.html), VE-821 (http://www.selleckchem.com/products/ve-821.html) and KU-60019 (http://www.selleckchem.com/products/KU-60019.html) were purchased from Selleckchem.

### Cells

HeLa-M cervical carcinoma cells were a gift from Andrzej Skladanowski (Gdansk, Poland). Parental A2780 and cisplatin resistant A2780/CP70 ovarian carcinoma cells were kindly provided by Robert Brown (Bearsten, UK), whereas IGROV1 ovarian carcinoma cells were provided by Alain Pierré (Croissy sur Seine, France). HeLa cells were grown in DMEM GlutaMAX™ (ThermoFisher Scientific) supplemented with 10% fetal bovine serum (Perbio Science). A2780, A2780/CP70 and IGROV1 were grown in RPMI 1640 medium (ThermoFisher Scientific) supplemented with 10% fetal bovine serum (Perbio Science). Media were supplemented with 100 units/ml penicillin and 100 μg/ml streptomycin (PanPharma). All cell lines were regularly tested for Mycoplasma contamination using Mycoplasma Detection Kit Myco Alert^®^ (Lonza).

### Antibodies

Antibodies directed againstP-Thr68-Chk2 (# 2661), Chk2 (clone 1C12, # 3440), P-Ser317-Chk1 (# 2344), Chk1 (clone 2G1D5, # 2360), P-Ser1981-ATM (clone 10H11.E12, # 4526) and RPA32 (clone 4E4, # 2208) were purchased from Cell Signaling Technology (Ozyme, Saint Quentin en Yvelines, France). Antibodies against phospho-Thr21-RPA32 (# ab61065) and MDC1 (# ab11169) were from Abcam while the H2AX (# 07-627) and γ-H2AX (# 05-636) -directed antibodies were purchased from Millipore (Lake Placid, NY). Antibodies against BRCA1 (# sc-6954) and RAD51 (# sc-8349) were from Santa Cruz Biotechnology. HRP (horseradish peroxidase) and fluorescent dye-conjugated antibodies were obtained from Jackson ImmunoResearch (Bar Harbor, ME).

### Viability assays

Cellular viability was determined by the MTT (methylthiazolyldiphenyl-tetrazolium bromide) assay as described previously [57]. Briefly, cells were exposed to the indicated concentrations of trabectedin or lurbinectedin for five doubling times. For drug combinations, cells were pre-incubated for 1 hour with checkpoint abrogators, followed by co-incubation with trabectedin or lurbinectedin for five doubling times. All values are averages of at least three independent experiments, each done in duplicate.

### Immunoblotting

HeLa cells were incubated with trabectedin or lurbinectedin for 1 hour and post-incubated in drug free medium for up to 6 hours at 37°C. Cells were then washed in PBS and lysed in lysis buffer (0.5% NP40, 20 mM Tris/HCl pH 8, 1 mM EDTA, 150 mM NaCl, 1 mM PMSF, 1 μM leupeptin, 1 μM aprotinin, 1 mM orthovanadate and 1 mM DTT) as described [58]. Proteins were resolved on SDS/PAGE (10 or 15%) and blotted onto nitrocellulose membranes (Bio-Rad). Membranes were saturated by TBST-milk [50 mM Tris/HCl (pH 8.0), 150 mM NaCl, 0.5% Tween 20 and 5% dehydrated skimmed milk] and the antigens were revealed by immunolabeling. Antigens were detected using an enhanced chemiluminescence kit (Bio-Rad) using the Chemidoc system (Bio-Rad).

### Immunofluorescence and microscopy

HeLa cells were incubated with the indicated concentrations of trabectedin or lurbinectedin for 1 hour at 37°C and post-incubated for 6 or 24 hours. For drug combinations, cells were exposed for 1 hour to trabectedin or lurbinectedin in the presence or absence of checkpoint abrogators followed by 24 hours in the presence or absence of checkpoint abrogators alone. Immunofluorescence experiments were carried out as described previously [59]. When indicated, coverslips were washed twice in PBS and resuspended in ice-cold CSK-lysis buffer (100 mM NaCl, 3 mM MgCl_2_, 1% Triton X100, 50 mM HEPES pH 7.4 and 300 mM sucrose) and kept at 4°C for 5 minutes before fixation in 4% paraformaldehyde (Electron Microscopy Sciences, Hatfield, PA, U.S.A). The antigens were revealed by using the indicated primary antibodies. DNA was counterstained by DAPI for fluorescent microscopy (# H-1200, Vector Laboratories) and TO-PRO-3 iodide for confocal analysis (# T3605, ThermoFisher Scientific). Images were collected using a BX61 fluorescent microscope and cell F imaging software (Olympus) or an inverted LEICA TCS SP2 confocal microscope. Fluorescence intensities were measured by MetaMorph software (Universal Imaging Corporation, Downingtown, PA). The background over noncellular regions was subtracted. At least 100 cells were analyzed per sample. Values represent the averages of at least three independent experiments.

### Chromosome spread

HeLa cells were exposed for 1 hour to the indicated concentrations of trabectedin or lurbinectedin in the presence or absence of checkpoint abrogators. Cells were then washed with PBS and post-incubated for 24 hours in the presence or absence of checkpoint abrogators. Chromosome spreads were prepared as described previously [60]. Briefly, cells were first treated with colchicine (0.2 μg/mL) for 90 minutes at 37°C, washed with PBS and incubated for 20 min at 37°C in hypotonic conditions (56 mM KCl). Cells were then fixed twice for 10 min at room temperature by acetic acid and methanol (1:3). After fixation, cells were dropped onto microscopy slide and DNA counterstained with DAPI. Images were collected using a BX61 microscope and cell F imaging software (Olympus). One hundred metaphases per treatment condition were evaluated. Cells presenting more than five chromosome breakages were considered as abnormal mitotic cells. The total number of cells in mitosis or in interphase was counted on each microscopy slides and the fraction of mitotic cells (mitotic index) was determined.

## SUPPLEMENTARY FIGURES


