# Supplementary material for: Dual inhibition of ATR and ATM potentiates the activity of trabectedin and lurbinectedin by perturbing the DNA damage response and homologous recombination repair
Source: Oncotarget. 2016 Mar 23;7(18):25885–901. doi: 10.18632/oncotarget.8292 (PMC5041952; doi:10.18632/oncotarget.8292)
Supplement: Supplementary file 1 [file oncotarget-07-25885-s001.pdf]

SUPPLEMENTARY FIGURES

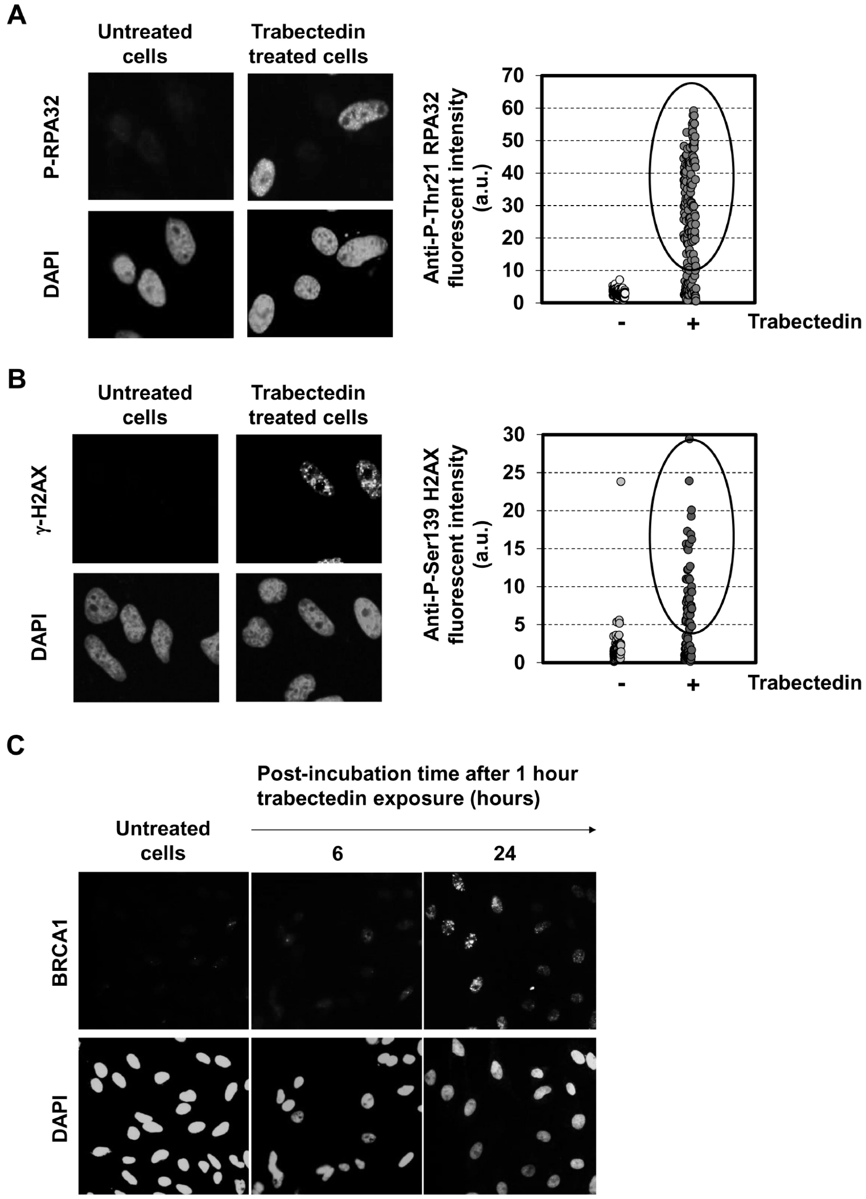

Supplementary Figure S1: Trabectedin induces phosphorylation of RPA32 and H2AX that is followed by focalization of BRCA1. A. HeLa cells were mock-treated or exposed to 20 nM trabectedin for 1 hour followed by 6 hours post-incubation in drug-free media. Cells were then fixed and processed for immunolabeling with an antibody directed against Thr21-phosphorylated RPA32. Left panel shows a typical image of Thr21-phosphorylated RPA32 obtained after exposure to trabectedin (trabectedin-treated cells) or no drug (untreated cells). The right panel shows the fluorescence intensities in individual cells as quantified by Metamorph analysis. The fluorescence intensities are indicated in arbitrary units (a.u.). At least 100 cells were analyzed for each condition. **B.** HeLa cells were mock-treated or exposed to 20 nM trabectedin for 1 hour followed by 6 hours post-incubation in drug-free media. Cells were then fixed and processed for immunolabelling with an antibody directed against Ser139-phosphorylated H2AX. Left panel shows a typical picture of Ser139-phosphorylated H2AX immunolabeling obtained after cells exposure to trabectedin (trabectedin treated cells) or no drug (untreated cells). The right panel shows the fluorescence intensities in single cells as quantified by Metamorph analysis. The fluorescence intensities are indicated in arbitrary units (a.u.). At least 100 cells were analyzed for each condition. **C.** HeLa cells were either untreated or incubated for 1 hour with trabectedin (20 nM) followed by 6 or 24 hours of post-incubation in drug-free media. Cells were then pre-permeabilized with ice-cold CSK-lysis buffer, fixed and immunolabeled with a BRCA1-directed antibody.

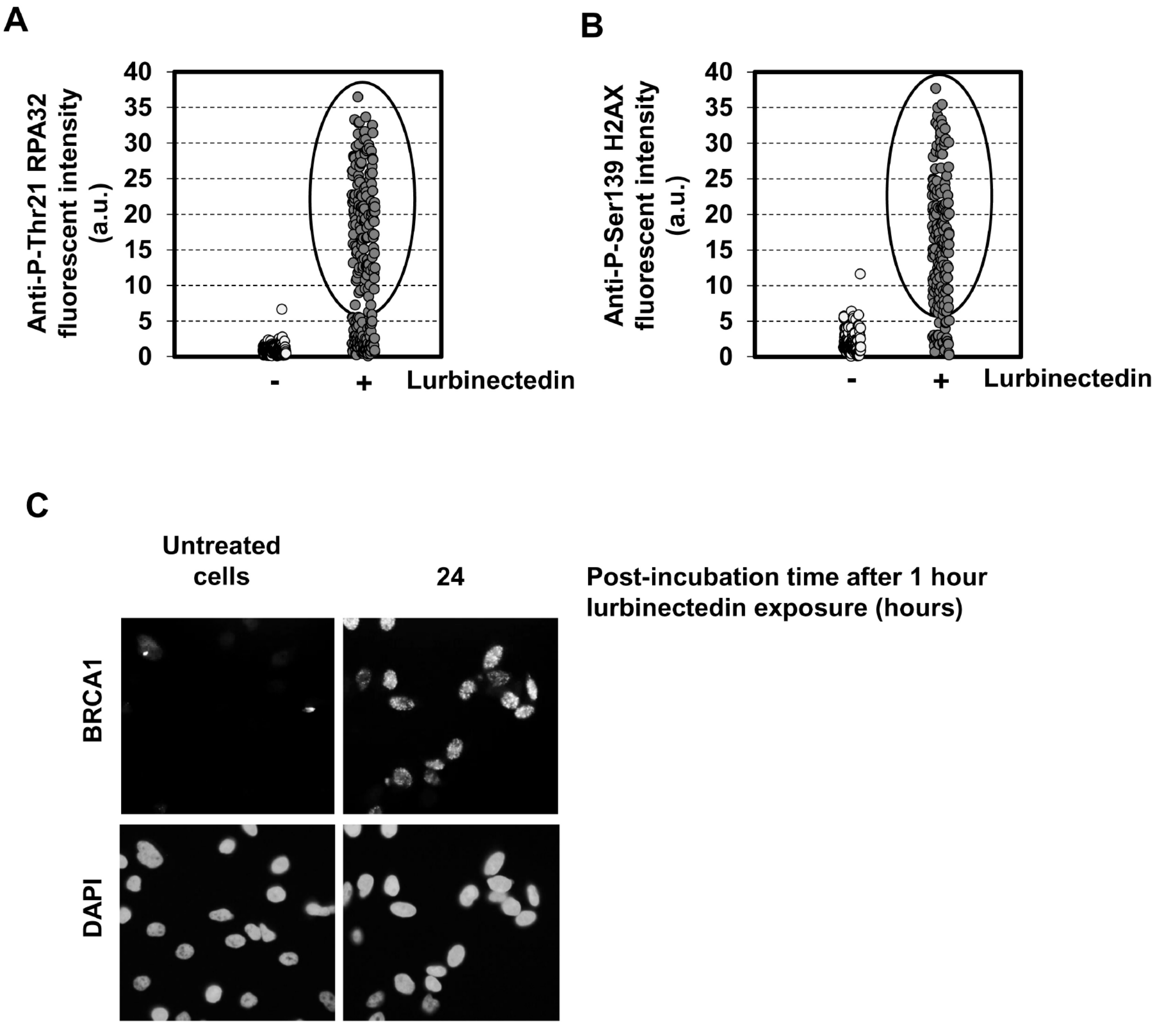

Supplementary Figure S2: Lurbinectedin induces phosphorylation of RPA32 and H2AX that is followed by focalization of BRCA1. A. HeLa cells were mock-treated (-) or exposed to 20 nM lurbinectedin (+) for 1 hour followed by 6 hours post-incubation in drug-free media. Cells were then fixed and processed for immunolabeling with an antibody directed against Thr21-phosphorylated RPA32. The fluorescence intensities in each single cell were quantified by Metamorph analysis and indicated in arbitrary units (a.u.). At least 100 cells were analyzed for each condition. **B.** HeLa cells were mock-treated (-) or exposed to 20 nM lurbinectedin (+) for 1 hour followed by 6 hours post-incubation in drug-free media. Cells were then fixed and processed for immunolabelling with an antibody directed against Ser139-phosphorylated H2AX. The fluorescence intensities in individual cells were quantified by Metamorph analysis and are indicated in arbitrary units (a.u.). At least 100 cells were analyzed for each condition. **C.** HeLa cells were either untreated or incubated for 1 hour with lurbinectedin (20 nM) followed by 24 hours post-incubation in drug-free media. Cells were then pre-permeabilized with ice-cold CSK-lysis buffer, fixed and immunolabeled with a BRCA1-directed antibody.

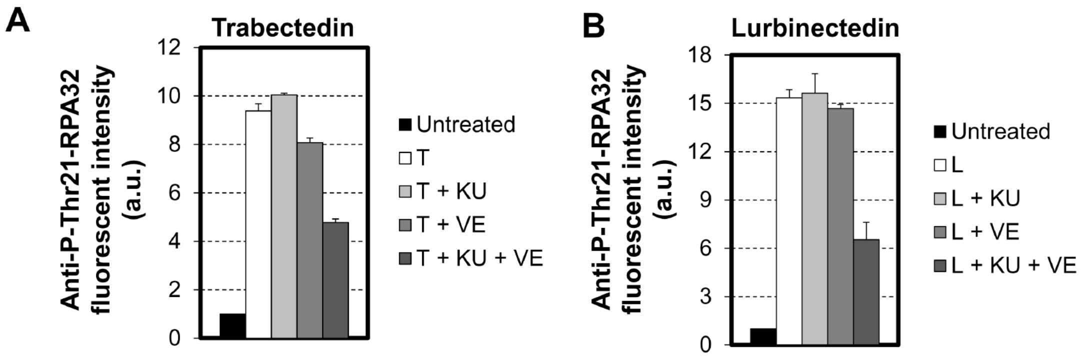

Supplementary Figure S3: Effect of checkpoint abrogators on the phosphorylation status of RPA32. A. HeLa cells were exposed to 10 nM trabectedin for 1 hour in the absence (T, white column) or presence of 2 μM KU-60019 (T + KU, light grey column), 1 μM VE-821 (T + VE, medium grey column) or [2 μM KU-600019 + 1 μM VE-821] (T + KU + VE, dark grey column). This was followed by 24 hours post-incubation in the absence (T, white column) or presence of 2 μM KU-60019 (T + KU, light grey column), 1 μM VE-821 (T + VE, medium grey column) or [2 μM KU-600019 + 1 μM VE-821] (T + KU + VE, dark grey column). Cells were then fixed and immunolabeled with a Thr21-phosphorylated RPA32 directed antibody. Untreated cells were used as a negative control. The fluorescence intensities in individual cells were quantified by Metamorph analysis and are indicated in arbitrary units (a.u.). At least 100 cells were analyzed for each condition. Values represent the averages of at least two independent experiments and data are represented as mean +/- SD. **B.** Same as above, except that cells were treated with lurbinectedin.
